# Evaluating the uptake and effects of the computerized decision support system NHGDoc on quality of primary care: protocol for a large-scale cluster randomized controlled trial

**DOI:** 10.1186/s13012-014-0145-5

**Published:** 2014-10-17

**Authors:** Marjolein Lugtenberg, Gert P Westert, Dennis Pasveer, Trudy van der Weijden, Rudolf B Kool

**Affiliations:** Scientific Institute for Quality of Healthcare (IQ healthcare), Radboud university medical center, P.O. Box 9101, Nijmegen, 6500 HB The Netherlands; School for Public Health and Primary Care (CAPHRI), Department of Family Medicine, Maastricht University, P.O. Box 616, Maastricht, 6200 MD The Netherlands

**Keywords:** Clinical decision support, Clinical practice guidelines, Primary care, Process of care, Patient outcomes

## Abstract

**Background:**

Computerized decision support systems (CDSSs) are increasingly used to improve quality of care. There is evidence for moderate to large effects from randomized controlled trials (RCTs), but evidence on their effectiveness when implemented at a national level is lacking. In the Netherlands, the Dutch College of General Practitioners (NHG) initiated their successful guideline program already 30 years ago. NHGDoc, a CDSS based on these NHG guidelines, covering multiple disease areas for general practice, was developed in 2006 with the aim to improve quality of primary care. In this paper, a protocol is presented to evaluate the uptake and effects of NHGDoc.

**Methods:**

A cluster RCT will be conducted among 120 general practices in the Netherlands. Eligible general practices will be randomized to receive either the regular NHGDoc decision support modules (control arm) or the regular modules plus an additional module on heart failure (intervention arm). The heart failure module consists of patient-specific alerts concerning the treatment of patients with heart failure. The effect evaluation will focus on performance indicators (e.g., prescription behavior) as well as on patient outcomes (e.g., hospital admissions) relevant in the domain of heart failure. Additionally, a process evaluation will be conducted to gain insight into the barriers and facilitators that affect the uptake and impact of NHGDoc.

**Discussion:**

Results of this study will provide insight in the uptake and impact of a multiple-domain covering CDSS for primary care implemented by a national guideline organization to improve the quality of primary care. Whereas the trial focuses on a specific domain of care—heart failure—conclusions of this study will shed light on the functioning of CDSSs covering multiple disease areas for primary care, particularly as this study also explores the factors contributing to the system’s uptake and effectiveness.

**Trial registration:**

Clinical trials NCT01773057

**Electronic supplementary material:**

The online version of this article (doi:10.1186/s13012-014-0145-5) contains supplementary material, which is available to authorized users.

## Background

Clinical practice guidelines aim to improve quality of care, but their implementation in practice remains a challenge. Despite considerable efforts in improving guideline implementation, several reviews have shown that guidelines are only moderately effective in changing clinical practice [1,2]. A study in the US showed that only about half of the patients received care as recommended in the guidelines [3]. Similarly, in other countries, adherence to the guidelines among physicians is found to be suboptimal as well [4,5].

Computerized decision support systems (CDSSs) are one of the tools that can be used to improve the uptake of guidelines in practice. By linking characteristics of individual patients to a computerized medical knowledge base, they can provide patient-specific recommendations to healthcare providers during patient care [6]. To the extent that CDSSs are guideline-driven, i.e., if the content is directly derived from clinical practice guidelines, they have the potential to increase physicians’ adherence to guidelines and ultimately lead to improved patient outcomes.

Whereas CDSSs are increasingly being used in various healthcare settings, evidence is mainly retrieved from small-scale academic-driven experiments among selected groups of innovators. Definitive evidence on their effectiveness in large-scale practice-driven use of CDSSs remains to be established. Some studies have shown that CDSSs can improve medical practice, e.g., [6-9]. In a recent series of six systematic reviews [10], it was found that CDSSs improved the process of medical care in 52%–64% of studies across all six reviews. However, only 15%–31% of those reviews that evaluated patient outcomes showed a positive impact on patients’ health [11-17].

Moreover, CDSSs seem to have added value as a tool for improving quality of care when focusing on specific behaviors (e.g., drug dosing) [6,7] within well-defined areas of care. A large share of medical care, however, is delivered by primary care practitioners (PCPs), particularly in the Netherlands [18]. PCPs work in generic settings and are confronted with a variety of diseases. This setting necessitates CDSSs covering multiple disease areas. Thus far, little is known on the effectiveness of CDSSs in settings in which PCPs are exposed to various alerts within multiple domains of care.

In the Netherlands, the Dutch College of GPs (NHG) initiated their successful guideline program already 30 years ago [19]. In 2006, NHGDoc, a CDSS based on these NHG guidelines and covering multiple disease areas for general practice, was developed as a collaborative effort between the Dutch College of GPs (NHG) [19] and ExpertDoc [20], a private enterprise. The content of NHGDoc is directly derived from the NHG guidelines—the national prevailing guidelines for general practice. NHGDoc is gradually being implemented at a national level and is currently—at the onset of the trial—integrated into two out of eight electronic health record systems (EHRSs) used in Dutch general practice, covering about 25% of all general practices in the Netherlands.

The aim of the study outlined in this protocol is to evaluate the uptake and effectiveness of the CDSS NHGDoc on the quality of primary care in the Netherlands. More specifically, our study’s aims are as follows:

1. To assess the effects of the CDSS NHGDoc on relevant performance indicators for the process of medical care
2. To assess the effects of the CDSS NHGDoc on patient outcomes
3. To gain insight into the determinants that affect the uptake and impact of NHGDoc

## Methods/design

### Study design

A two arm-cluster (before and after) RCT will be conducted with a follow-up period of 12 months. Randomization will take place at a practice level, i.e., general practices will be randomized to the control arm or the intervention arm rather than individual PCPs. This will be the preferred unit of allocation as the intervention (i.e., the NHGDoc module on heart failure) can only be implemented at the level of the practices. Moreover, this is done to avoid possible contamination between PCPs working within the same practice yet being allocated to different study groups [21].

This study has been designed and will be reported in accordance with the CONSORT statement [22,23] and its extension regarding cluster RCTs [24] (see Additional file 1).

### Setting

The study will be conducted within the Dutch primary care setting. In the Netherlands, there are approximately 5,000 general practices in which nearly 11,000 GPs are delivering care [25]. Almost all GPs are members of the Dutch College of General Practitioners (NHG). Since the late 1980s, the NHG has produced over 100 national clinical practice guidelines for general practice [19]. These NHG guidelines cover the vast majority of conditions and diseases frequently seen in general practice. Currently, eight of these guidelines have been integrated into NHGDoc, i.e., cardiovascular risk management, asthma/COPD, diabetes mellitus type II, thyroid disorders, viral hepatitis and other liver diseases, atrial fibrillation, and subfertility.

Additional to GPs, approximately 3,000 practice nurses (PNs) are delivering care in approximately 60% of the Dutch general practices. They are mainly responsible for regular checks of the chronically ill such as cardiovascular and asthma/COPD patients [26].

NHGDoc was developed in 2006 as a collaborative effort between the NHG [19] and ExpertDoc [20]. As the NHG is the owner of the system and is an organization highly appreciated among GPs, it was decided to introduce the name ‘NHGDoc’ to the CDSS. NHGDoc is at the time of recruiting practices for the study integrated into two (MicroHIS X and Promedico-ASP) out of eight EHRSs used in Dutch general practice. These two systems cover about 25% of all Dutch general practices.

### Participants

All general practices in the Netherlands that use either the EHRS MicroHIS X or Promedico-ASP and thus have NHGDoc at their disposal, will be invited to participate in the study (*n* = approximately 1,100). To invite general practices to participate in the study, a comprehensive recruitment plan will be executed. All general practices will be approached by ordinary post mail signed by the NHG as well as an email signed by the EHRS providers (MicroHIS X and Promedico-ASP) and their user associations (Orego and Atlas). Additionally, announcements about the evaluation study will be placed in several relevant Dutch journals, websites, newsletters, and through social media (i.e., Facebook, Twitter, and LinkedIn). In all messages, reference will be made to the website of the evaluation study (www.nhgdoc-evaluatie.nl) which presents information about the study as well as an online registration form for general practices. To enable blinding of participants and contrast between groups, the study is purposefully presented as a before-after study without a concurrent control group, rather than as a before-after RCT. Therefore, the participants will not be aware of the fact that they will be allocated at random to two different conditions.

In order to participate in the study, each general practice should fill out the online registration form. The applicant is asked to consent on behalf of all practice staff (GPs and PNs) that could potentially use NHGDoc. This is necessary as the intervention and data collection can only be implemented at the level of the practices. We will, however, be able to distinguish between different users in analyzing the study results.

### Intervention and comparisons

#### Control arm: regular NHGDoc decision support

General practices assigned to the control arm receive the regular NHGDoc decision support. These practices receive decision support with respect to all modules (NHG guidelines) that—at the onset of the trial—have been integrated into NHGDoc (see ‘Description of NHGDoc and its regular modules (control arm)’ section).

#### Description of NHGDoc and its regular modules (control arm)

NHGDoc is a CDSS integrated within the EHRS and based on the NHG guidelines, the prevailing guidelines for general practice in the Netherlands. It provides GPs and PNs evidence-based and patient-specific advices during consultation in terms of patient data registration, drug prescription, and management.

At the time of the onset of the trial, NHGDoc covered the following NHGDoc modules: cardiovascular risk management, asthma/COPD, diabetes mellitus type II, thyroid disorders, viral hepatitis and other liver diseases, atrial fibrillation, and subfertility. For each NHG guideline, key recommendations have been selected based on relevance of disease burden, room for improvement, and possibility to translate or normalize the recommendation into if-then rules. This selection of key recommendations is approved by representative experts of the guideline committees. Subsequently, the selected key recommendations are digitized into NHGDoc.

When the GP or PN opens a patient file in the EHRS, anonymous patient and performance data are sent to the NHGDoc server. The patient and performance data are compared to the digitized guideline recommendations and in the case of a discrepancy between current and advised care, an alert will be sent back to the GP or PN.

#### Intervention arm: regular modules plus additional module on heart failure

General practices allocated to the intervention arm will receive the same decision support modules as the control arm (see ‘Description of NHGDoc and its regular modules (control arm)’ section), extended with the NHGDoc module on heart failure, which will be activated at the onset of the trial (see ‘Basic elements/key recommendations of the NHGDoc module on heart failure (intervention arm)’ section). The reason we chose heart failure to be the subject of the trial was first of all of pragmatic nature; heart failure was one of the few modules that were to be implemented into NHGDoc in the year of the onset of the trial. In addition, we chose heart failure as it is a relatively common condition for which relatively large improvements (in terms of increasing or decreasing mortality and morbidity) are to be expected if healthcare providers adhere to the treatment guidelines, compared to other conditions [27-31].

#### Basic elements/key recommendations of the NHGDoc module on heart failure (intervention arm)

The NHGDoc module on heart failure is directly derived from the NHG guideline on heart failure [32]. It consists of three types of alerts:

1. Alerts on heart failure in terms of registering the following patient data:
  clearance of creatinine;
  (serum) creatinine;
  (serum) potassium;
  (serum) sodium;
  patient weight;
  heart (frequency);
  blood pressure (systolic and diastolic).
2. Alerts on heart failure in terms of prescribing (or adjusting the dose of) the following drugs:
  (lis)diuretics;
  ACE-inhibitor;
  AII antagonist;
  potassium suppletion;
  beta-blocker;
  aldosterone antagonist;
  digoxin.
  For example: Stop prescribing ACE-inhibitor because of the reduced clearance of creatinine and the increased serum potassium and check these values.
3. Alerts on heart failure in terms of (paying attention to) the following management aspects such as:
  stop prescribing medication such as calcium antagonist, thiazolidinedione, disopyramide or a combination of ACE-inhibitor and AII antagonists;
  consider adjusting the dosage of medication when hypertension is increasing;
  consider use of NSAID;
  consider weight policy, e.g., discuss weight reduction and consider increasing the dosage of diureticum if weight increases with two kilogram or more within a short period of time;
  consider temporary restriction of fluids;
  advice to give up smoking;
  consider sleeping research and;
  consider referral to a cardiologist or nephrologist.

### Study measures

#### Outcomes

To select outcome measures for our evaluation study, we first identified previously validated indicators for heart failure [33]. Subsequently, we consulted an expert panel of general practitioners as well as specialists in the area of heart failure to select the outcome measures that are clinically most relevant for our study and that are suitable for quantitative evaluation. Outcomes will be measured during the year before the start of the intervention (baseline) and in the first year following the start of the intervention (follow-up). This resulted in the following primary and secondary outcome measures:

#### Primary outcome measures (per practice, per year):

- ➢ Prescribing of ACE-inhibitors/angiotensin II

Percentage of consultations in which a patient with heart failure was prescribed ACE-inhibitors/angiotensin II.

- ➢ Prescribing of beta-blockers

Percentage of consultations in which a patient with heart failure was prescribed beta-blockers.

- ➢ Prescribing of diuretics

Percentage of consultations in which a patient with heart failure was prescribed diuretics.

#### Secondary outcome measures:

- ➢ Use of the system NHGDoc

Number of requests sent and alerts received for all patients; number of requests sent and alerts received for heart failure patients; percentage of opened alerts compared to the number received alerts; percentage of opened alerts compared to the number received alerts for heart failure patients; mean delivery time for an alert; number of practices that switched any module off; for each module, the number/percentage of practices with at least one user who switched the module off; and number of practices with at least one user who switched the module heart failure off (intervention group only).

- ➢ Patient data registration

Percentage of consultations in which for a patient with heart failure, relevant patient data have been registered: clearance of creatinine (<12 mnd); (serum) creatinine (<12 mnd); (serum) potassium (<12 mnd); (serum) sodium (<12 mnd); patient weight (<6 mnd); heart (frequency) (<6 mnd); blood pressure (systolic and diastolic) (<6 mnd).

- ➢ Hospital admissions

Number of hospital admissions of patients with heart failure.

- ➢ All cause mortality

Number of patients with heart failure that died in the hospital.

### Sample size

The minimum required number of general practices was based on the two main primary outcome measures: prescribing of ACE-inhibitors/angiotensin II and prescribing of beta-blockers. The anticipated improvement was based on literature [27,28] as well as on consultation of experts in the field (ACE-inhibitor change from 63%–69%; beta-blockers from 59%–65%). As for beta-blockers, to detect this difference, we will need at least 122 general practices completing the trial with 80% power at a type I error risk (*α*) of 5%, taking an estimated intra-cluster correlation of 0.1 into account [34]. With this number of practices, the study will also be able to detect the difference for prescribing of ACE-inhibitors (minimum number of practices =116). We do not, however, expect to be able to detect an effect on hospital admissions and hospital mortality with this number of practices.

### Randomization

**Figure 1:**
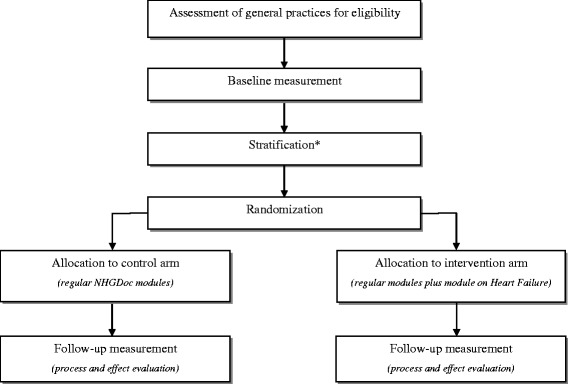
**Study flow diagram.** *Stratification will be based on the type of EHRS.

Eligible general practices will be randomly allocated to either the intervention arm or the control arm, stratified by type of EHRS (MicroHIS X or Promedico-ASP) (see Figure 1). Practices will be the unit of randomization. Before randomization, the practices will be clustered at the level of practice addresses (practice AGB code and zip code) to avoid GPs and practices that are located within the same building being designated to different study groups. Randomization will be central and computer-generated, executed by an independent person (one of the members of the research team without any COI towards allocation). Dedicated software will be used to generate a randomization scheme with an equal number of control and intervention practices.

### Blinding

General practices and the staff working within these practices will be blinded to group allocation. They will not be informed about being allocated to either the intervention group or the control group (see ‘Participants’ section). They are even unaware of participating in an intervention trial with a concurrent control group. However, as the intervention arm will, at the onset of the trial, receive decision support on heart failure (directly available to all intervention practices at one preset moment in time) without the usual communication about the implementation of a new module, these practices may suspect heart failure to be the subject/topic of the trial. Therefore, the extent to which the blinding was really successful will be checked in a survey, which is part of the process evaluation of the trial.

Patients will also be blinded to group allocation as they are not aware and are not likely to become aware of the trial (design). Given the fact that patients are not being exposed to experimental interventions and that all patient data used in our evaluation study are anonymized, we are not obliged to inform patients about the use of these data, nor do we have to offer them an opt-out option. And finally, researchers will also be blinded to the group allocation as the randomization variables will be labeled with the letters A and B (rather than I and C) for the control and intervention group by a third independent researcher.

### Data collection

To investigate the effectiveness of NHGDoc, data will be collected from two sources: the NHGDoc server and Dutch Hospital Data.

#### NHGDoc server

For the largest part of this study, existing data collection methods from the NHGDoc alert service that have been developed by ExpertDoc to generate CDSS alerts for NHGDoc will be used. These are all automated data extractions from electronic patient records available in the EHRS. The NHGDoc alert service only uses anonymized patient data, and data are only streamed and not stored. For this evaluation study, however, it is necessary to save the anonymous data for the follow-up period of a year. Therefore, ExpertDoc, the company that manages the data, will send the NHGDoc data on a monthly basis, secured with a log in code, to IQ healthcare. NHGDoc data include all alerts, and the anonymous patient data that generate the alerts.

#### Dutch Hospital Data

To be able to detect the effects of NHGDoc on patient outcomes, we also use data from Dutch Hospital Data (DHD), a foundation that collects and manages data of Dutch hospitals. Based on the practice codes (practice AGB codes) of the participating practices in both arms, DHD selects relevant data with respect to hospital admissions and mortality of patients that are registered with ICD-code I50 of the ICD 10 and sends them, after anonymizing the dataset, to IQ healthcare.

### Analysis

To assess the effects of NHGDoc in terms of improving the quality of primary care, the scores on outcomes measures during the follow-up period will be compared between both study arms adjusted for baseline findings. To take into account potential clustering of effects within practices, multilevel models will be used.

The primary outcome measures are the percentage of consultations in which a patient with heart failure is prescribed 1. ACE-inhibitors/angiotensin II, 2. beta-blockers, and 3. diuretics. To assess the effects of NHGDoc on each of these variables, multilevel linear regression analysis will be performed. To assess the effects of NHGDoc on mortality, logistic regression analysis will be used, adjusting for severity of illness at hospital admission and comorbidity as measured by the Charlson Comorbidity Index. Both patient-level and practice-level intercept estimates will be used to account for potential correlation of measurements within patients and within general practices. We do not use the provider level estimates in our analyses as the difference between number of practices and providers will be too small due to solo practices. Moreover, patients may visit different GPs within the same practice during the year making this distinction less relevant as well.

### Process evaluation

Aside from the effect evaluation, a process evaluation will be conducted to gain insight into the barriers and facilitators that affect the system’s uptake and impact [35]. The actual exposure of the PCPs to the intervention as well as their experience with the intervention may have affected the results of the study and will be investigated. Results of the process evaluation will be used to improve (the implementation of) NHGDoc.

Data will be collected among participants in the intervention and control arm in three different ways. First, some variables measuring exposure to the intervention (e.g., not functioning of the server, deliberately turning off the heart failure module or particular areas within this module) will be collected by the NHGDoc server.

Second, three focus groups will be conducted among NHGDoc users (eight to ten GPs and PNs per group) to gain insight into their experiences with CDSSs in general and NHGDoc in particular. In each focus group, barriers and facilitators will be discussed using a topic list. Participants will be recruited by sending a direct email and reminders to all participating practices. If necessary, additional announcements will be placed on relevant websites and through social media (i.e., Facebook, Twitter, and LinkedIn).

And finally, an electronic questionnaire will be sent to all participating practices at the end of the study period to triangulate the findings of the study. Aside from quantitatively assessing the perceived barriers and facilitators to using CDSSs in general and NHGDoc in particular, attention will be paid to the exposure to and experiences with the specific heart failure module. Also, the intended ‘blinding’ in terms of the content of the intervention as well as the group allocation will be checked among participants.

Exposure to and experiences with NHGDoc will be analyzed descriptively. Data from the focus groups will be audiotaped, verbatim transcribed, and independently analyzed by two researchers using content analysis with the software program AtlasTi7.0.

### Ethical considerations

In the Netherlands, studies involving human subjects need to undergo a medical ethics review if they are subject to the Medical Research Involving Human Subjects Act (WMO). The study protocol was assessed by the Medical Ethics Committee (CMO) (of district Nijmegen/Radboud) and declared that no further ethical approval was required.

Moreover, the Dutch College of General Practitioners (NHG) set up a privacy college for the use of data from NHGDoc in order to monitor whether all data related to NHGDoc are used appropriately. Therefore, the study protocol of our evaluation study was checked and approved by this college. Also, the members from the college have the option throughout the trial to check on whether the data were encrypted and delivered as agreed on in the protocol.

## Trial status

At the time of manuscript submission, the trial is closed to recruitment and follow-up. No data cleaning or analysis has been executed prior to the submission of this manuscript.

## Discussion

This paper describes the protocol of a cluster RCT, which aims to evaluate the effectiveness of a multiple-domain covering CDSS for primary care implemented by a national organization. By means of a large-scale 1-year trial, it will be investigated whether providing guideline-driven and patient-specific advices to healthcare providers during consultation leads to improved process of medical care and ultimately to improved patient outcomes. Additionally, insight will be gained into the barriers and facilitators perceived by users that may affect NHGDoc’s uptake and impact.

The effectiveness of CDSSs in primary care has been evaluated in previous studies [6-17]. However, the CDSS being subject of this trial exhibits some unique features. First, NHGDoc is a guideline-driven CDSS for primary care, which is being implemented by a national organization. Whereas most CDSSs incorporate evidence-based recommendations, in a few of them, the whole content is directly derived from a set of national prevailing clinical practice guidelines developed by a national independent and respected organization. Moreover, the system NHGDoc is also owned and implemented by this independent professional organization. A CDSS such as NHGDoc that is owned and implemented by an independent professional national organization might be more effective compared to CDSSs for which this link is not established.

Another distinguishing feature of NHGDoc is its focus on the generic setting of primary care. Most CDSSs have been developed to target specific behaviors (e.g., preventive measures such as vaccinations, appropriate drug dosing) within well-defined areas of care (e.g., intensive care unit). NHGDoc is developed for a generic setting and integrates multiple alerts within multiple domains of care or disease areas. Although CDSSs have proven to be successful in focusing on specific behaviors within well-defined areas of care, very little is known [36,37] on how systems designed for and used in a generic primary care setting will function. This trial will shed some light on this issue.

Although NHGDoc is a multiple-domain covering CDSS, the design of the study does not allow us to differentiate in the effects between the various domains of care. The intervention in our trial consists solely of decision support in the area of heart failure. Nevertheless, our study does provide insight into the extent that decision support on heart failure is effective in a setting in which decision support is provided for several domains of care. Moreover, the process evaluation in our study will also provide insight in terms of the uptake and effectiveness of decision support as part of a multiple domain-covering CDSSs as well as its contributing/factors.

A limitation of our study design is that we do not have a ‘real’ control group, consisting of healthcare providers not using NHGDoc at all. Although the control arm does not receive decision support in the area of heart failure, it does receive decision support with respect to the regular NHGDoc modules that had already been implemented at the onset of the trial. This may suggest a selection bias with only GPs and PNs with a positive attitude towards CDSSs participating in the trial. However, both the control and intervention arm consist of general practices that have NHGDoc at their disposal because it is integrated into their EHRS, rather than that they have deliberately chosen for it. As a consequence, it is unlikely that only highly motivated GPs and PNs using NHGDoc frequently participate in our study. Moreover, if there is any bias in terms of motivation among participating GPs, we expect it to be equally distributed between the intervention and control arm, as a result of the randomization process of the trial.

This study will shed light on the uptake and effectiveness of CDSSs covering multiple disease areas in primary care, which is owned and implemented by a national guideline organization. By also exploring factors contributing to the system’s uptake and effectiveness, insight will be provided in how such systems should be designed and implemented in order to function well in a generic primary care setting. Results will not only be of interest to developers and implementers of CDSSs but also to guideline implementers using CDSSs to improve the uptake of guidelines and ultimately patient outcomes in primary care.

## Additional file

Additional file 1: Table S1. CONSORT 2010 checklist of information to include when reporting a cluster randomised trial. **Table S2.** Extension of CONSORT for abstracts to reports of cluster randomised trials.
